# Environmental Monitoring of *Legionella* in Hospitals in the Campania Region: A 5-Year Study

**DOI:** 10.3390/ijerph20085526

**Published:** 2023-04-14

**Authors:** Annalisa Lombardi, Tonia Borriello, Elvira De Rosa, Fabiana Di Duca, Michele Sorrentino, Ida Torre, Paolo Montuori, Ugo Trama, Francesca Pennino

**Affiliations:** 1 Department of Public Health, University “Federico II”, Via Sergio Pansini N° 5, 80131 Naples, Italy; 2 General Directorate of Health, Campania Region, Centro Direzionale C3, 80143 Naples, Italy

**Keywords:** *Legionella*, environmental monitoring, Campania region, hospital, residual chlorine, temperature

## Abstract

*Legionella* is a pathogen that colonizes soils, freshwater, and building water systems. People who are most affected are those with immunodeficiencies, so it is necessary to monitor its presence in hospitals. The purpose of this study was to evaluate the presence of *Legionella* in water samples collected from hospitals in the Campania region, Southern Italy. A total of 3365 water samples were collected from January 2018 to December 2022 twice a year in hospital wards from taps and showers, tank bottoms, and air-treatment units. Microbiological analysis was conducted in accordance with the UNI EN ISO 11731:2017, and the correlations between the presence of *Legionella* and water temperature and residual chlorine were investigated. In total, 708 samples (21.0%) tested positive. The most represented species was *L. pneumophila* 2–14 (70.9%). The serogroups isolated were 1 (27.7%), 6 (24.5%), 8 (23.3%), 3 (18.9%), 5 (3.1%), and 10 (1.1%). Non-*pneumophila Legionella* spp. represented 1.4% of the total. Regarding temperature, the majority of *Legionella* positive samples were found in the temperature range of 26.0–40.9 °C. An influence of residual chlorine on the presence of the bacterium was observed, confirming that chlorine disinfection is effective for controlling contamination. The positivity for serogroups other than serogroup 1 suggested the need to continue environmental monitoring of *Legionella* and to focus on the clinical diagnosis of other serogroups.

## 1. Introduction

*Legionella* is an aerobic, non-spore-forming, and Gram-negative pathogen [1], which was discovered in 1976 in Philadelphia following an outbreak of cases of pneumonia in a hotel [2]. Over 65 species belong to this genus [3], about 20 of which can cause disease in humans [4]. Moreover, *Legionella pneumophila* includes 15 serogroups, and serogroup 1 is the most dangerous to humans. Thus, the attention focused on this serogroup is high [4]. Furthermore, previous studies demonstrated human infections with serogroup 3 [5,6,7], serogroup 9 [7,8], and serogroup 6 [7,9].

Favourable factors to the growth of this bacterium are temperatures between 25 °C and 45 °C (but ranges between 5.7 °C and 63.0 °C can determine its survival); stagnant water [10], in which *Legionella* in amoebae copiously reproduces [11]; low flow [12]; pH values between 5.5 and 9.2; biofilm and protozoa [2]; inorganic elements (iron, zinc and potassium) and organic and inorganic compounds [13]; existence of water systems of old facilities [14]; dead branches in complex water structures [15]; low total chlorine levels [16]; and low free residual chlorine levels [17]. In addition, man-made disasters [18], natural disasters, flooding [19], water system interruptions, changes in disinfection methods of water systems, and water network breaks [11] are risk factors for *Legionella* growth.

Construction activities (such as demolition, repressurization, excavation, underground utility connections, commissioning at building opening, and water efficiency challenges) have been associated with healthcare-associated *Legionella* infections and deaths [20].

Hospitals need a water safety plan (WSP) to control *Legionella* proliferation, which includes the implementation of safety measures to ensure the water quality of facilities during demolition and construction activities and reduce the risk of exposure of patients [21]. Furthermore, hospitals need a WSP not only during construction or demolition activities. In fact, the World Health Organization (WHO) in 2004 recommended the creation of WSPs by all water suppliers: each of them should engage a group of water experts to assess the risks associated with water exposure, develop and implement strategies to prevent damage to public health, and evaluate the strategies’ effectiveness [22]. Such a plan aims to minimize colonization of *Legionella* from the source of water supply to the devices in contact with users [23].

Chlorine-based disinfection is the most common active measure used worldwide, and in Italy, against *Legionella* growth and spread in building water systems [24,25]. Levels of free residual oxidant (FRO), within 0.20 ppm and 4.0 ppm in potable water, have been correlated with reduced risks of growth and spread of disease outbreaks [26].

The presence of *Legionella* can be detected in soil and water sources, such as showers, hot tubs, air-conditioning systems [3], cooling towers, whirlpools, baths, fountains, ice machines, medical equipment such as aerosols from respiratory devices, eye-wash stations, dental units [27], hot-water recirculation systems [28], hot- and cold-water systems, faucets [29], heater–cooler units, and heater units for cardiac procedures [30].

*Legionella* infection occurs through inhalation or aspiration of droplets released from contaminated water [3]. In addition, potting soil can also transmit the bacterium, but the mechanism still remains unknown [27]. People who are most affected by *Legionella* infections are smokers [31], alcohol abusers [32], males, those of advanced age [33], and people with previous diseases, such as acquired immunodeficiency syndrome, hematologic malignancy [34], and diabetes mellitus [35]. Consequently, it is necessary to monitor the presence of *Legionella* in hospitals as these are places where immunocompromised patients reside [36]. In healthcare settings, the presence of *Legionella* has been linked to contaminated water reservoirs, cooling towers and air-conditioning systems [37,38,39]. The occurrence of *Legionella* spp. in the water distribution systems of hospitals and healthcare facilities is a possible concern for hospital populations, due to the vulnerable nature of patients admitted to specific wards, including intensive care, hematology, cardiology, hemodialysis, and pulmonology [40].

The objective to be achieved in healthcare facilities is to minimize colonization by *Legionella* or, in the case of facilities housing immunocompromised individuals, its total absence (not detectable with the analytical method used) [41].

*Legionella* infection may have several consequences: it may cause Legionnaires’ disease (LD) (a severe pneumonia), Pontiac fever (a flu-like condition), or it may remain asymptomatic. Because of the lack of specific symptoms and the absence of severity associated with this entity, Pontiac fever is often undiagnosed and under-notified [42].

There is a difference between patients who are hospitalized for LD [43] and others who acquire the infection in hospital due to susceptible medical conditions [44]. LD can be the cause of CAP (community-acquired pneumonia) and pneumonias that are acquired in situations other than travel, domestic environments, and hospitals, and it can be the cause of up to 30% of these types of pneumonias that require hospitalization [43]. *Legionella* infection can also be acquired in hospitals (hospital-associated infection): individuals most at risk from such an infection are those with prior situations that render them vulnerable, such as immunodeficiency, stem cell or organ transplantation, and obstructive pulmonary disease [44].

*Legionella* infections are becoming a public health problem due to their incidence and costs [45]. In order to prevent and control *Legionella* infections sourced from the colonization of water systems, many countries have developed guidelines or regulations. Several organizations, including the American Society of Heating, Refrigerating and Air-Conditioning Engineers (ASHRAE), the WHO, the Centers for Medicare and Medicaid Services (CMS), and the Centers for Disease Control and Prevention (CDC), recommend the creation of water management programs aimed at preventing the growth and spread of *Legionella* [46].

In the COVID-19 era, it was reported that 20% of patients had a *Legionella* co-infection during hospitalization [47]. COVID-19 patients have an increased risk for both hospitalization and residual lung impairment [48].

In 2021, the number of Italian individuals affected by Legionellosis was 2726, and its incidence was 46.0 cases per million population [49]. The incidence increased compared to 2020, when the value was 34.3 cases per million population. Of the 2726 notified cases, 83.6% had a community origin, 9.4% were associated with travel, 3.7% had a nosocomial origin, 3.1% were associated with closed communities (nursing homes for elderly people, and healthcare or rehabilitation facilities), and 0.2% had another exposure (prison or communities). Infections of nosocomial origin increased from 68 to 102 cases from 2020 to 2021 [49]. However, *Legionella* infections are underestimated, and it is reported that less than 5% are diagnosed [50].

Legionellosis is a condition that can be avoided if the bacterium is not present in the environment, so monitoring and preventing its presence is important [51]. Environmental monitoring of *Legionella* is an approach performed on several occasions in hospitals in Italian regions by Deiana et al. [40], Ditommaso et al. [52], Vincenti et al. [53], De Giglio et al. [54], Laganà et al. [14], Arrigo et al. [55], Pasquarella et al. [56], and Torre et al. [57]; in non-hospital facilities by Totaro et al. [58,59], Sabatini et al. [60], and De Filippis et al. [61]; and in both hospital and non-hospital facilities by Felice et al. [62], Leoni et al. [63], and Mazzotta et al. [64]. Moreover, several studies were also conducted using air samples by Montagna et al. [65,66,67].

To our knowledge, very few studies performed environmental monitoring of *Legionella* in the Campania region, Southern Italy. In particular, a study by Torre et al. monitored the bacterium in 50 hospitals in the Campania region during the period 2008–2014 [39]. As the new Italian National Guidelines [41] were published in 2015, it was decided to conduct this study to assess the effectiveness of the application of the new Guidelines in preventing *Legionella* colonization in water systems. Thus, the aim of this study was to analyze hospital environmental monitoring of *Legionella* in the Campania region over the 5-year study period. In detail, the purposes of the study were (i) to evaluate the presence of *Legionella* in tested water samples; (ii) to estimate the prevalence of species and of individual *L. pneumophila* serogroups; and (iii) to assess the influence of several parameters, such as temperature and free residual chlorine, on the presence of this bacterium.

## 2. Materials and Methods

### 2.1. Study Area and Hospital Characteristics

**Figure 1:**
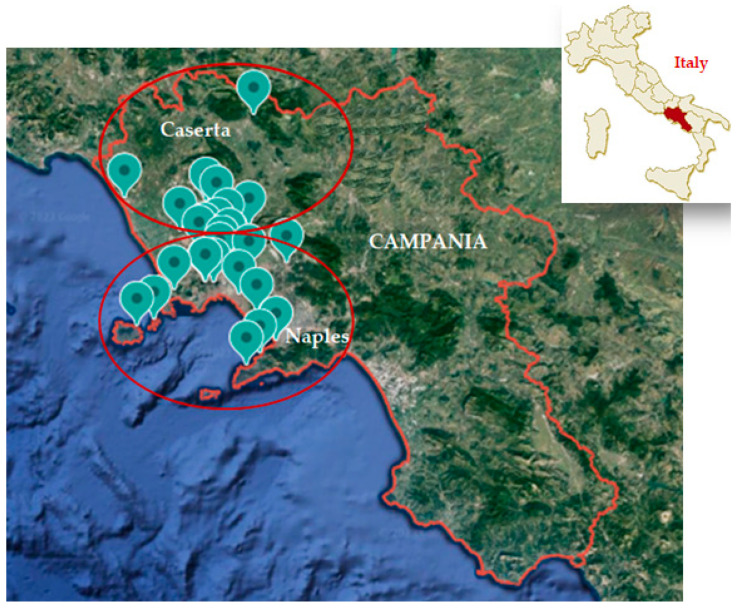
Study area map.

The water sample collection was conducted from January 2018 to December 2022 in two provinces of the Campania region, Southern Italy (Naples and Caserta) (Figure 1). The samples were collected in hospitals which ask us to routinely perform *Legionella* testing every six months as part of WSPs as required by the Italian regulatory system for validation of a well-maintained building water system. The water samples were collected from 26 hospitals, all of which possessed the following characteristics: the presence of a single building, construction between 1970 and 1980, the presence of a maximum of five floors, and the presence of the number of beds served ranging between 20 and 200. These hospitals all have WSPs in place to respond to positive samples. We monitored 26 hospitals compared to the 50 hospitals in the study by Torre et al. [39], because some of the 50 hospitals were excluded since monitoring had not been carried out for all the years of the study, or these facilities were not comparable to the others (in terms of number of beds served, number of buildings, number of floors, construction period, or water treatment disinfection methods).

Water provided to the hospitals (which contains free chlorine as residual drinking water disinfectant) comes from the public supply system of the cities of Naples and Caserta, and it reaches the hospitals via a single pipeline. The hospital sites within the present study included buildings hosting patients considered at increased risk (Medicine, Pneumology, Geriatrics, Surgeries, etc.) [41] over the study period, inclusive of the time during the COVID-19 pandemic.

Samples were collected every six months, as required by the Italian National Guidelines for hospitals with this type of wards. If a sample tested positive, according to the Italian National Guidelines, the sampled point was decontaminated and re-sampled 1, 3, and 6 months later (these mandatory follow-up lab samples and corresponding data were excluded from the present study’s analysis, as the purpose of the work was to monitor *Legionella* during routine monitoring activities). Therefore, we included the initial positive samples and excluded the follow-up samples. The water treatment disinfection methods used in these hospitals are based on the application of hypochlorite.

### 2.2. Sample Collection

We chose the sampling locations described in the Italian National Guidelines [41]. In addition, this study used similar sampling locations (i.e., faucets, showers, and tank bottoms) within the building premise plumbing system of the study by Torre et al. [39]. Our study, in addition to the previous sampling locations, also analyzed samples collected from the cold-water circuit, as mentioned in the new Guidelines.

In detail, for each hot-water system, as described in the Italian National Guidelines [41], the following sampling locations were carried out: supply, recirculation, and tank bottoms, with at least 3 representative points (furthest in the water distribution and coldest). Tank bottom refers to a hot- or cold-water storage tank. For each cold-water system, the following sampling locations were carried out: tank bottoms, with at least 2 representative points (furthest in the water distribution and hottest). In addition, water was collected from air-treatment units (ATUs). Following the observation of the positivity rate for *Legionella*, it was decided to increase the sampled points. The COVID-19 pandemic prompted us to further increase the number of samplings, and with its end, we decided to decrease the number of sampled points. Of the total of 3365 water samples, 2065 originated from manual taps and showers (1485 from hot water and 580 from cold water), 780 from tank bottoms (in particular, 520 from hot water and 260 from cold water), and 520 from ATUs. The water samples were collected during the day (later in the morning, when the hospital was already in action). There were no point-of-use (POU) filters at any point of sampling.

According to the Italian National Guidelines, 1 L of water was collected by the laboratory staff in sterile polyethylene bottles enriched with 0.01% sodium thiosulfate to neutralize chlorine action [60]. All samples were collected without flushing and flaming or disinfecting at the point of discharge to simulate the common use of water, i.e., the exposure of a user. The water samples were univocally identified on a spreadsheet at the time of collection. At sampling, water temperature and residual chlorine were measured by our laboratory staff. Water temperature (expressed in °C) was obtained using a calibrated thermometer (TFA Digitales Einstichthermometer, TFA-Dostmann, GmbH & Co. KG, Wertheim-Reicholzheim, Germany), and free residual chlorine (expressed in mg/L) was monitored using a colorimetric diethyl-p-phenylenediamine (DPD) method (MQuant; Merck, Darmstadt, Germany). In detail, the test was based on a semi-quantitative measurement of free chlorine by visual comparison of the color of the measurement solution and a set of colors contained in a color card comparator.

The samples were transported, divided between hot- and cold-water samples at room temperature, and protected from light.

Calibrations (kits and equipment) and microbiological analysis were performed in our laboratory, which is accredited according to the ISO 17025 and periodically performs proficiency tests.

No samples were damaged during transport or showed a suspect appearance, such as different coloring or the presence of sediment or soil. Therefore, all samples collected were included and analyzed in the study.

### 2.3. Microbiological Analysis and Identification

The microbiological analysis was conducted within 2 hours after the collection of the samples, in accordance with the UNI EN ISO 11731:2017 [55,64]. Briefly, the samples were filtered on polycarbonate membrane filters with a pore size of 0.2 µm (Sartorius). The membrane was kept in 10 mL of the original water sample and vortexed. A total of 200 µL of water previously treated at (50 ± 1) °C for (30 ± 2) min. and the same volume of untreated water were inoculated on Petri plates containing *Legionella* Agar Base (Oxoid) medium and being supplemented with *Legionella* Growth Supplement (BCYE) (Oxoid) and *Legionella* Selective Supplement (GVPC) (Oxoid). The plates were incubated at (36 ± 2) °C with 2.5% CO_2_ and under a humid atmosphere. After 10 days, the presence or absence of colonies was evaluated. The presumed *Legionella* colonies were cultured both on BCYE agar and BCYE agar without L-cysteine (BCYE-cys agar). The growth of colonies on the BCYE agar and not on the BCYE-cys agar suggested *Legionella* positivity of the samples. Determination of species and serogroups was conducted by the latex agglutination test (Oxoid) and the anti-*Legionella pneumophila* monovalent serum (Biogenetics). The colony counts were reported in terms of CFU (colony-forming units)/L.

The test results were sent to the hospitals.

### 2.4. Statistical Analysis

A descriptive statistical analysis was performed as previously described [39]. It included, for bacterial load values of positive samples, detection of geometric mean (Log_10_ CFU/L), standard deviation, median, percentile range, and interquartile range [39]. This analysis was conducted using Microsoft Excel.

Normality tests were conducted using the Shapiro–Francia test to check for data distribution. The non-parametric Mann–Whitney U test was used to determine the connections between the presence of *Legionella* and residual chlorine (expressed in mg/L) and water temperature (expressed in °C) [68]. The statistical results were interpreted at the level of significance *p* < 0.05. The χ^2^ was calculated using Doornik–Hansen test. Multiple linear regression analysis (MLRA) was used to confirm the results of the Mann–Whitney U test. The statistical calculations were performed using the STATA MP v14.0 statistical software program (College Station, TX, USA).

## 3. Results

### 3.1. Positivity of Analyzed Samples

**Figure 2:**
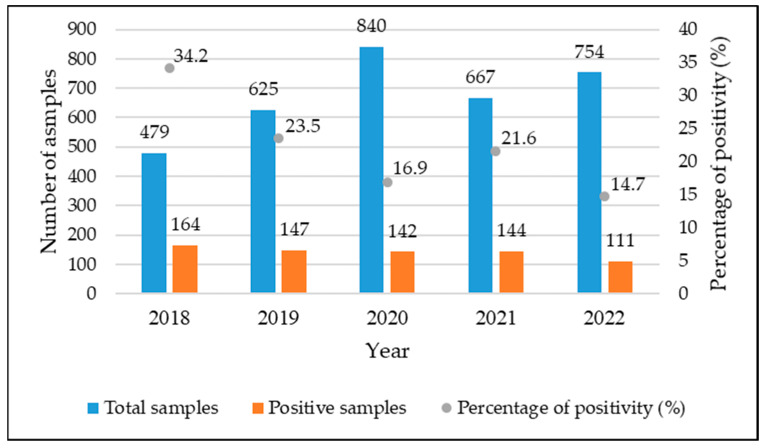
Number of collected samples, number of positive samples, and positivity percentages by sampling year.

During the 5-year study period, the laboratory analyzed a total of 3365 water samples. Among these samples, positivity for *Legionella* was detected in 708 samples, representing 21.0% of the total. The number of samples per year, along with the number of positive samples per year, is shown in Figure 2 and Table 1. Specifically, the number of positive samples decreased from 164 (34.2%) to 111 (14.7%).

Table 2 shows the number of total samples, the number of positive samples, and the percentage for different sampling locations. Of the total number of cold-water samples (840), 37 (4.4%) tested positive. In particular, 11 originated from tank bottoms and 26 from taps and showers. In the hot-water samples, out of the 2005 total samples, 652 (32.5%) were positive. A total of 143 samples came from tank bottoms and 509 from taps and showers. In the ATU samples, a positivity rate of 3.7% was observed (19 positive samples out of a total of 520).

### 3.2. Distribution of Species and Serogroups among Positive Samples

**Figure 3:**
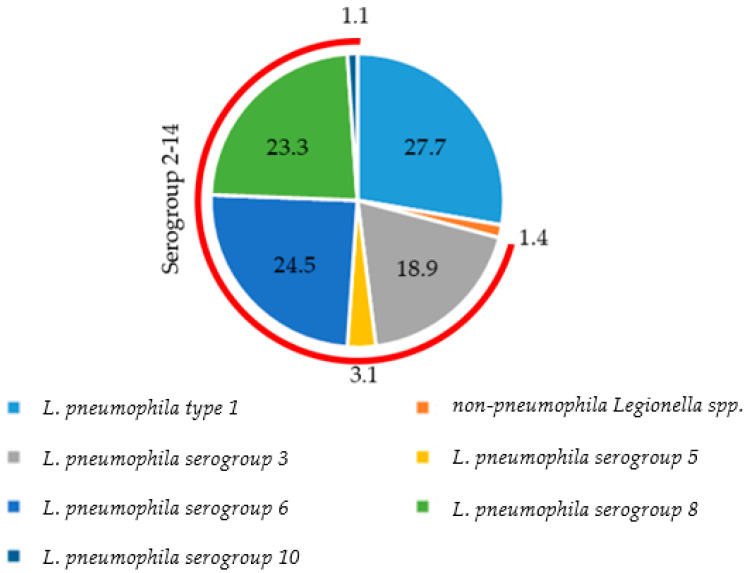
Percentage of positivity of each *Legionella* species and serogroup during the five-year study period (2018–2022).

Figure 3 exhibits the percentage of positivity of *Legionella* species and serogroups in the years 2018–2022. In detail, *L. pneumophila* was the most represented (98.6% versus 1.4% of non-*pneumophila Legionella* spp.). Serogroups 2–14 had a positivity percentage of 70.9%, and serogroup 1 has a positivity percentage of 27.7%. Among serogroups 2–14, out of the total number of isolated species and serogroups, the most represented were serogroups 6 (24.5%), 3 (18.9%), and 8 (23.3%). Serogroups 5 and 10, on the other hand, were detected at a rate of 3.1% and 1.1%, respectively (Figure 3).

In addition, in 27 samples of the 708 positive samples, the simultaneous presence of two species or serogroups was found. Particularly, out of these 27 samples, 22 samples tested positive for both serogroups 1 and 3 (3.1%), 1 sample tested positive for serogroups 1 and 6 (0.1%), 2 samples tested positive for serogroups 1 and 8 (0.3%), 1 sample tested positive for serogroups 1 and 10 (0.1%), and 1 sample tested positive for serogroup 5 and non-*pneumophila Legionella* spp. (0.1%).

### 3.3. Water Temperature and Residual Chlorine

Of the 3365 analyzed samples, 520 were taken from the ATUs in which the temperature and residual chlorine parameters were not carried out, while the remaining 2845 samples were divided into hot-water samples (≥26.0 °C, n. 2,005) and cold-water samples (≤25.8 °C, n. 840), with a mean temperature of 37.5 °C (range of 11.0–91.0 °C) for all the samples collected.

Table 3 shows different ranges of temperature and, for each interval, the number of analyzed samples, the number of positive samples, the minimum *Legionella* concentration, the maximum *Legionella* concentration, and the geometric mean of the positive samples. The majority of *Legionella* positive samples were found in the temperature ranges of 26.0–30.9 °C, 31.0–35.9 °C, and 36.0–40.9 °C (57.4%, 46.9%, and 48.8%). The percentage of positive samples decreased with increasing temperatures (from 48.8% for the temperature range of 36.0–40.9 °C to 19.4% for temperatures ≥56.0 °C).

Regarding the bacterial concentration of the positive samples, a minimum value corresponding to 1.70 Log_10_ CFU/L was observed at all temperature ranges, while the highest maximum value was observed at the temperature range of 46.0–50.9 °C (4.36 Log_10_ CFU/L).

In addition, the minimum value of the geometric mean was 2.81 Log_10_ CFU/L in temperatures ≤20 °C, and it increased with increasing temperature, up to the range of 26.0–30.9 °C (3.10 Log_10_ CFU/L). For the temperature range of 41.0–45.9 °C and above, there was a decrease in the geometric mean value as temperature increased. At temperatures higher than 62 °C, no positivity for *Legionella* was observed.

### 3.4. Statistical Analysis

The results of the statistical analysis carried out to examine the bacterial load values of the positive samples are shown in Table 4. This table reports the number of total samples, the number of positive samples and percentage of positivity, the geometric mean (Log_10_ CFU/L), the standard deviation, the median, the percentile range, and the interquartile range. Moreover, the results show the normality of all data collected, and the Doornik–Hansen test for multivariate normality provides a χ^2^ = 1.09 × 10^5^.

The lowest *Legionella* concentration value recorded during the analysis was 1.70 Log_10_ CFU/L in all years, while the highest value was 4.36 Log_10_ CFU/L in 2019.

**Table 4:** Number of total samples, number of positive samples and percentage of positivity, geometric mean (Log_10_ CFU/L) of positive samples, and descriptive statistics. Descriptive statistical analysis was conducted for the positive samples (N = 708).

Year	Pos./Tot. (%)	Mean (±SD ^1^)	Min	25th Percentile	Median	75th Percentile	Max
2018	164/479 (34.2)	3.02 ± 0.72	1.70	2.54	3.12	3.63	4.28
2019	147/625 (23.5)	2.71 ± 0.80	1.70	2.00	2.60	3.35	4.36
2020	142/840 (16.9)	2.82 ± 0.74	1.70	2.18	2.81	3.38	4.30
2021	144/667 (21.6)	2.96 ± 0.73	1.70	2.40	3.02	3.62	4.23
2022	111/754 (14.7)	3.14 ± 0.58	1.70	2.78	3.20	3.62	4.04

^1^ SD: standard deviation.

The results of the MRLA (Table 5) indicated that no statistically significances were recorded for the water samples’ temperature (*p*-value = 0.526), in contrast with residual chlorine (*p*-value < 0.05).

Furthermore, the MRLA results revealed the statistically significant correlation between residual chlorine and *Legionella* concentration, in which was negative, as suggested by the t-value (−6.19) and *p*-value (<0.05).

**Figure 4:**
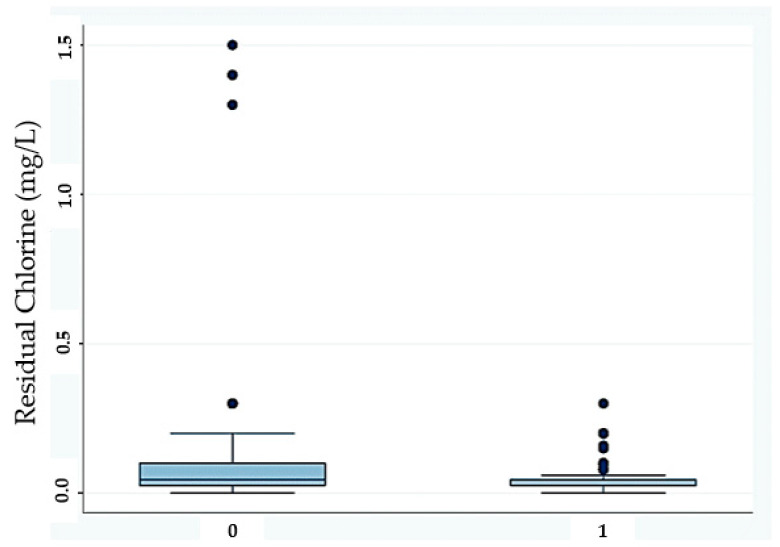
Boxplot showing the influence of chlorine residue (expressed in mg/L) on the presence of *Legionella*. 0 = No *Legionella* detected (N = 2156), and 1 = *Legionella* detected (N = 689).

The boxplot regarding the influence of residual chlorine is shown in Figure 4.

## 4. Discussions

*Legionella* is a bacterium that colonizes soils, freshwater, and building water systems [51]. The aim of this paper was to analyze the prevalence of *Legionella* and its individual species and serogroups in hospitals of the Campania region over the period 2018–2022 and to assess the effectiveness of the Italian National Guidelines of 2015 in preventing *Legionella* colonization in water systems. In addition, the purpose was to test whether there was a relationship between the presence of *Legionella* with two variables, residual chlorine and water temperature.

In this study, we observed a 21.0% positivity rate. The positivity rate for *Legionella* decreased from 2018 (34.2%) to 2022 (14.7%). The number of positive samples gradually decreased, and this explains the decrease in the positivity percentage for this microorganism (Figure 2). Deiana et al. [40], on the other hand, monitored the presence of *Legionella* from 2010 to 2020 in a university hospital in the Sardinia region, Italy. Regarding the period 2018–2020, they also observed a decrease in the percentage of *Legionella* positivity in this region.

The authors posit that the decrease in *Legionella* positivity over the course of the study is likely attributed to the hospitals receiving the study’s results and performing environmental hazard controls. The COVID-19 pandemic in 2020 and 2021 decreased attention to environmental *Legionella* prevention, leading to an increase in *Legionella* positivity in 2021. This increase was not observed in 2020 because the increase in hospitalization led to the need to implement hygiene rules (e.g., hand washing) [69], which could have promoted water flow and prevented stagnation. Stagnation is known to be responsible for the disappearance and poor stability of residual disinfectant and microbial growth of *Legionella* [70]. Moreover, the monitored hospitals were all in constant activity during the pandemic. The pandemic also directed our attention to new locations to monitor for *Legionella*: monitoring more locations in hospitals meant preventing *Legionella* infections in patients.

The percentage of positivity observed at the different sampling locations was 19.7% for tank bottoms, 25.9% for taps and showers, and 3.7% for ATUs. This study, in general, used similar sampling locations (i.e., faucets, showers, and tank bottoms) within the building premise plumbing system of the study by Torre et al. [39], conducted in the years 2008–2014 in hospitals in the Campania region, which showed a percentage of positivity of 27.2% for tank bottoms, 31.9% for taps and showers, and 4.0% for ATUs. The authors supposed that this decrease could be explained by the publication of the Italian National Guidelines in 2015 and the implementation of a WSP by hospitals, which resulted in the collaboration of different personnel (laboratory staff, technical office, water disinfection staff, etc.) in order to ensure water safety. In addition, the highest percentage of *Legionella* detection was observed in taps and showers, which are a source of exposure for patients (taps and showers can generate aerosols containing the bacterium that can be inhaled) [71].

Out of a total of 840 cold-water samples (temperature range of 11.0–25.9 °C), 37 (4.4%) tested positive. The presence of *Legionella* in cold-water samples was also found in the study by Arvand et al., 2011 [72], who detected a percentage of positivity of 40% in cold-water samples (temperature range of 7–29 °C) [72]. Our results are not surprising, since out of the total number of cold-water samples taken (840), 458 (54.5%) showed a temperature between 21.0 and 25.9 °C, which are values near the range of temperatures that can create favorable conditions for *Legionella* multiplication.

The results indicated that *L. pneumophila* was the most isolated species. The serogroups 2–14 showed a positivity rate of 70.8% for the positive samples, which was similar to that found in Turkish hospitals (70.8%) by Yilmaz and Orhan [73], in Italian non-hospital facilities (71.7%) by Sabatini et al. [60], in hospital samples (68.7%) collected by Deiana et al. [40], and in Polish non-hospital samples (64.3%) collected by Stojek and Dutkiewicz [74].

Regarding the individual serogroups, the highest percentage of positivity was found for serogroup 1. This finding is congruent with what is found in clinical diagnosis, according to which the *L*. *pneumophila* type 1 is the serogroup that most commonly affects humans [7,75,76]. Amemura-Maekawa et al., in fact, isolated an 80.2% percentage of serogroup 1-positive samples in Japanese clinical specimens collected from 1980 to 2008 [77].

Regarding serogroups 2–14, the presence of serogroups different from serogroup 1 (6, 8, and 3) was the same as found previously [39]. Serogroups 3 and 6 were also among the serogroups isolated from humans in Italy during 1987 and 2009 [78], so these results had correspondence with what was previously reported in humans. These findings were also in agreement with the results determined by Perola et al., who, through a genotyping analysis, found an association between the isolates from two *Legionella* serogroup 5-positive patients residing for several days in a hospital and the environmental isolates from the hospital’s water supply [79].

Serogroups 3 and 6 were found in environmental water samples in several studies [54,80,81,82,83]. Particularly, serogroup 3 was isolated in hospitals in Iran [84], while Pignato et al. revealed the prevalence of this serogroup in Italian hospitals, which was sometimes associated with serogroup 6 [85]. Isolated serogroup 8 was reported by De Giglio et al. [54], Papadakis et al. [83], and Sakhaee et al. [84].

The presence of serogroups different from serogroup 1 suggests that attention should be focused on the clinical diagnosis of other serogroups, considering that the most widely used test for *Legionella* diagnosis in human specimens (the urinary antigen test) shows a low sensitivity for non-*L. pneumophila* type 1, resulting in underreporting of *Legionella* infections [76,86].

Regarding temperature, in our study, we observed that more hot-water samples were positive than cold-water samples (32.5% vs. 4.4%). This finding is consistent with that observed in the work by Stojek et al. conducted in Poland, who observed a higher frequency of *Legionella* isolation in hot-water samples (88.6%) [87]. In addition, the highest percentages of positive samples were found at temperatures ranging from 26.0 to 40.9 °C, which are temperatures corresponding to the optimal growth range of *Legionella*. For temperatures above this range, the percentage decreased. With regard to the bacterium’s concentrations, the highest values were found at a temperature range of 46.0–50.9 °C, and above this temperature range, these values decreased. In the study by Boppe et al. conducted in a hospital where the correlation between the concentration of *L. pneumophila* and the maximum water temperature at the point of use was investigated, a significant decrease in the bacterium’s concentrations was observed at temperatures ≥55 °C [88].

Our analysis revealed a *Legionella* concentration minimum value of 1.70 Log_10_ CFU/L in all years, and the highest value of 4.36 Log_10_ CFU/L was observed in 2019. In the study by Torre et al., the minimum and maximum values observed were 2.00 and 7.45 Log_10_ CFU/L [39]. On the other hand, in the study by Girolamini et al., the minimum and maximum concentration values observed in a hospital in the Emilia-Romagna region, Italy, which water was treated with a biocide, were between <1.70 and 5.80 Log_10_ CFU/L [89].

The Italian National Guidelines for the prevention and control of Legionellosis indicate the types of interventions required to be conducted in healthcare facilities, depending on the positivity rate, the *Legionella* concentration (expressed in CFU/L), and the presence or absence of clinical cases [41].

In addition, procedures must be performed to obtain non-detection of *Legionella* in air-treatment systems and water in wards housing profoundly immunocompromised and very high-risk patients (transplant centers, oncology, and hematology) and in the water for tank births.

If a sample tested positive, according to the Italian National Guidelines, the sampled point is decontaminated and re-sampled one, three, and six months later (these mandatory follow-up lab samples and corresponding data were excluded from the present study’s analysis).

Regarding the trends of *Legionella* concentration found, this study revealed a statistically significant negative correlation with residual chlorine. This finding was in agreement with D’Alessandro et al. [90], who observed a statistically significant reduction in positive samples following the increase in free chlorine in water. Furthermore, Totaro et al. found a moderate relationship between the presence of *Legionella* and a decrease in total chlorine concentration [58]. The same result was reported by Masaka et al., who found a weak statistically significant negative correlation with both *L. pneumophila* serogroups 1 and 2–14 [91]. A statistically significant negative correlation between *Legionella* concentration and total chlorine residual concentration was reported by Zhang et al. [92]. Moreover, in their study, Rafiee et al. argued that there was proportionality between residual chlorine content and the presence of *Legionella* [93]. The mechanism by which chlorine combats the presence of *Legionella* involves it interaction with the pathogen’s cell membrane [94]. This results in a dispersion of the cell’s macromolecules and subsequent modification of the cell’s chemical, physical, and biological processes [94].

Furthermore, no statistically significant correlation was found with water temperature, as reported by Masaka et al. [91] and Pierre et al. [46], and in contrast to the evidence found by Rakić et al. [68], who found a correlation with *L. pneumophila*. In addition, De Giglio et al. observed a weak correlation between *Legionella* concentrations and water temperature [95].

In summary, the results of the study confirm that chlorine disinfection is an effective method to control *Legionella*; this is in accordance with the results reported by Paranjape et al., who found that continuous application of chlorine inhibits the presence of *Legionella* in cooling towers, and by Orsi et al., who found that shock and continuous hyperchlorination significantly reduce the number of *Legionella* positive samples [16,96].

## 5. Limitations

The limitations of this study were that sampling covered only two provinces of the Campania region in the South of Italy. Thus, in the future, it would be interesting to expand the study to other provinces in order to have a complete overview of the situation in the study area. Moreover, the sampling covered only one type of facility. Consequently, a future goal could be to expand the analysis to other types of facilities, such as recreational centers, tourist sites, military bases, schools, and others, since it is possible to identify the bacterium in these structures [62]. In addition, only two variables were analyzed to evaluate the relationship with the presence of *Legionella* (water temperature and residual chlorine). Furthermore, we did not collect information about cases of *Legionella* infections in the monitored hospitals, and it would be interesting to investigate this aspect in future studies and assess whether there is a correlation between these two variables, as performed in the study by Perola et al. [79].

Finally, the circumstance that, in a high percentage of samples (79.0%), *Legionella* is not detected by the culture method does not mean that all samples are really negative for *Legionella*. There may be a percentage of samples that have viable but non-culturable (VBNC) *Legionella*, and the culture method cannot detect them. VBNC is a state of *Legionella* that can be established under unfavorable environmental conditions and retain virulence. Under favorable conditions and in free-living amoebae, the bacterium can resuscitate and become pathogenic again [97]. For example, a study conducted from 2010 to 2015 in an Italian university hospital subjected to continuous monochloramine treatment determined that 34.5% of the negative samples, as determined by the culture method, tested positive for the VBNC state. In addition, 18% of 22 tested samples were positive based on the resuscitation test [97]. This situation could occur in some of our negative samples. Future goals will include testing for the VBNC state.

This paper could be a starting point for future analyses, given the importance of environmental monitoring and the number of studies monitoring this bacterium in the Campania region in South of Italy.

## 6. Conclusions

Our environmental monitoring revealed bacterial contamination of water in this region, even though its presence decreased in percentage positivity from 2018 to 2022. Despite this decline, attention should not be reduced, especially in facilities housing immunocompromised individuals who are more susceptible to *Legionella* infection. The most represented species was confirmed to be *L*. *pneumophila*. Serogroups 2–14 were found at a high rate. Regarding total serogroups, the most represented was serogroup 1, followed by serogroups 6, 3, and 8. These results suggest the need for continuous environmental monitoring of *Legionella* and highlight the importance of focusing on the clinical diagnosis of serogroups different from serogroup 1 as well, since the most widely used test on human samples shows a high sensitivity only for this serogroup.

The highest concentration of positive samples was observed in the temperature range corresponding to the optimal growth temperature of the bacterium. In addition, the negative correlation between residual chlorine and the presence of *Legionella* confirmed that chlorine disinfection is an effective method for preventing *Legionella* contamination.

The implementation of a WSP, through the collaboration of different groups of professionals, is one of the main approaches to control *Legionella* contamination in hospitals and prevent infections.

## Figures and Tables

**Table 1:** Summary of the total number of samples collected during the 5-year study period (2018–2022) with the number of positive samples and related percentages.

Sampling Year	Total Number of Samples	Number of Positive Samples (%)
2018	479	164 (34.2)
2019	625	147 (23.5)
2020	840	142 (16.9)
2021	667	144 (21.6)
2022	754	111 (14.7)

**Table 2:** Total samples, positive samples, and percentages for different sampling locations.

Tank Bottoms		Taps and Showers		ATUs
Cold	Hot	Cold	Hot	
N. (%)	N. (%)	N. (%)	N. (%)	N. (%)
11/260 (4.2%)	143/520 (27.5%)	26/580 (4.5%)	509/1485 (34.3%)	19/520 (3.7%)

**Table 3:** Number of analyzed samples, number of positive samples, percentage of positive samples, minimum and maximum values, and geometric mean of *Legionella* concentration of positive samples for different ranges of temperature.

				*Legionella* Concentration (Log_10_ CFU/L)		
	Temperature (°C)	Analyzed Samples	Positive Samples (%)	Min	Max	Geometric Mean
Cold-water samples (n. 840)	≤20.9	382	19 (5.0%)	1.70	3.81	2.81
	21.0–25.9	458	18 (3.9%)	1.70	4.00	2.83
Hot-water samples (n. 2005)	26.0–30.9	54	31 (57.4%)	1.70	4.18	3.10
	31.0–35.9	179	84 (46.9%)	1.70	4.23	2.98
	36.0–40.9	240	117 (48.8%)	1.70	4.30	3.00
	41.0–45.9	558	170 (30.5%)	1.70	4.30	2.96
	46.0–50.9	549	153 (27.9%)	1.70	4.36	2.87
	51.0–55.9	296	72 (24.3%)	1.70	3.96	2.67
	≥56.0	129	25 (19.4%)	1.70	3.97	2.73

**Table 5:** Results of linear multiple regression.

	Unstandardized Coefficients		Standardized Coefficients	
	T	Standard Error	*t*	*p*-Value
Dependent Variable: Bacterial Concentration (CFU/L)				
Water Temperature (°C)	0.0005	0.0008	0.63	0.526
Residual Chlorine (mg/L)	−0.4844	0.0782	−6.19	<0.05

N = 2845.

## Data Availability

The datasets obtained and analyzed in the current study are available from the corresponding author on reasonable request.
